# Supplementary material for: A novel deep learning-based framework with particle swarm optimisation for intrusion detection in computer networks
Source: PLoS One. 2025 Feb 12;20(2):e0316253. doi: 10.1371/journal.pone.0316253 (PMC11819531; doi:10.1371/journal.pone.0316253)
Supplement: Attachment — Submitted filename: Response to Reviewers.docx [file pone.0316253.s001.docx]

**Original Manuscript ID:** PONE-D-24-38218

**Original Article Title:** “A Novel Deep Learning-based Framework with Particle Swarm Optimisation for intrusion detection in computer networks”

To: PLOS ONE Journal Editor

Re: Response to reviewers

Dear Editor,

We would like to thank you for allowing a resubmission of our manuscript, with an opportunity to address the reviewers’ comments. We also would like to thank the reviewers for their constructive comments to improve the current presentation of the paper.

We are uploading (a) our point-by-point response to the comments (below) (response to reviewers), (b) A marked-up copy of your manuscript that highlights changes made to the original version (revised manuscript with track changes), and (c) a clean updated/revised manuscript without tracked changes (manuscript).

Best regards,

Abdullah Asım YILMAZ

**Response to Reviewer 1**

**Author Response:** We would like to thank the reviewer for his/her positive and constructive comment on our manuscript. We addressed each of them in the revised manuscript.

**Reviewer#1, Concern # 1:** The motivation and contribution of this paper should be stated more clearly in the abstract to better understand from the beginning of the study. Authors are advised to be precise in the abstract, and structure your abstract as follows- 1) Background 2) Aim/Objective 3) Methodology 4) Results 5) Conclusion. Write 2-4 lines for each and merge everything in one paragraph (150-250 Words) without any subheading.

**Author Response:** Thank you for the comment we do agree and have made changes accordingly in the abstract of manuscript. In addition to the sentences previously added to the article, the updates made in the abstract section are shown in the Author Action section.

**Author Action:** Intrusion detection plays a significant role in the provision of information security. The most critical element is the ability to precisely identify different types of intrusions into the network. However, the detection of intrusions poses a important challenge, as many new types of intrusion are now generated by cyber-attackers every day. A robust system is still elusive, despite the various strategies that have been proposed in recent years. Hence, a novel deep-learning-based architecture for detecting intrusions into a computer network is proposed in this paper. The aim is to construct a hybrid system that enhances the efficiency and accuracy of intrusion detection. The main contribution of our work is a novel deep learning-based hybrid architecture in which PSO is used for hyperparameter optimisation and three well-known pre-trained network models are combined in an optimised way. The suggested method involves six key stages: data gathering, pre-processing, deep neural network (DNN) architecture design, optimisation of hyperparameters, training, and evaluation of the trained DNN. To verify the superiority of the suggested method over alternative state-of-the-art schemes, it was evaluated on the KDDCUP’99, NSL-KDD and UNSW-NB15 datasets. Our empirical findings show that the proposed model successfully and correctly classifies different types of attacks with 82.44%, 90.42% and 93.55% accuracy values obtained on UNSW-B15, NSL-KDD and KDDCUP’99 datasets, respectively, and outperforms alternative schemes in the literature.

**Reviewer#1, Concern # 2:** What are the main contributions of the paper in terms of novelty, technique used and observation? Since there are many Hybrid-Intrusion detection system available in literature, what are the issues this paper address is missing in the paper.

**Author Response:** Thank you for the comments. This study makes five major contributions to the literature, unlike other studies in the literature. Firstly, it introduces a novel hybrid deep-learning-based intrusion detection method. Secondly, the PSO method is used to optimise the hyperparameters, thus enhancing the performance of the model. Thirdly, the method is evaluated on three extensive intrusion detection datasets to ensure its effectiveness and applicability. Fourthly, the proposed approach incorporates a new hash layer that combines three pre-trained models, which distinguishes it from conventional deep learning approaches that use only one model. Lastly, the method can significantly reduce the feature space and achieves superior accuracy compared to other known methods, representing a valuable advancement in intrusion detection research.

The proposed deep learning technique was assessed on UNSW-B15, NSL-KDD and KDDCUP’99 datasets, and the performance of the proposed hybrid model was initially compared with each model in isolation. The findings demonstrated that our approach could successfully classify intrusion with high values for the F1-score, accuracy, recall and precision metrics. Next, our model was evaluated on the NSL-KDD dataset using confusion matrices, and it was shown to generate better results than the alternative models for all types of intrusion apart from U2R. Finally, a comparison between our architecture and state-of-the-art alternatives was performed, and results revealed that the proposed method was superior to the other methods.

**Author Action:** In addition to the previously added sentences in manuscript, the Introduction and Conclusion Sections of manuscript has been updated in manuscript as shown below. Details were shown at a marked-up copy of our manuscript (revised manuscript with track changes).

For the Introduction Section:

Over the past few years, significant numbers of researchers have adopted deep learning techniques across diverse fields, such as graph-based applications, hand gesture recognition, enhancement of driving safety, identification of human actions, recognition of facial emotions, speech recognition, natural language processing, malware classification, medical applications, and object detectio1. The achievements of deep learning techniques have attracted the interest of scholars in the field of intrusion detection, and research in this field has been undertaken to tackle the shortcomings of existing intrusion detection techniques and to elevate the overall effectiveness of models. However, the development of a resilient system remains challenging. Robust systems are of the utmost importance for reducing feature spaces and computational complexity, surmounting the constraints imposed by time, resources, and hardware, and augmenting the accuracy rates of IDSs. Hence, this study presents a new framework for detecting intrusions that exploits the capabilities of deep learning methods. The objective is to build a hybrid system that improves intrusion detection's effectiveness and precisionconstruct a hybrid system that enhances the efficiency and accuracy of intrusion detection.

This study makes five major contributions to the literature. Firstly, it introduces a novel hybrid deep-learning-based intrusion detection method. Secondly, the PSO method is used to optimise the hyperparameters, thus enhancing the performance of the model. Thirdly, the method is evaluated on three extensive intrusion detection datasets to ensure its effectiveness and applicability. Fourthly, the proposed approach incorporates a new hash layer that combines three pre-trained models, which distinguishes it from conventional deep learning approaches that use only one model. Lastly, the method can significantly reduce the feature space and achieves superior accuracy compared to other known methods, representing a valuable advancement in intrusion detection research.

For the Conclusion Section:

Our work's primary contribution is a novel deep learning-based hybrid architecture that combines three well-known pre-trained network models in an optimized manner while using PSO for hyperparameter optimization. The proposed deep learning technique was assessed on UNSW-B15, NSL-KDD and KDDCUP’99 datasets, and the performance of the proposed hybrid model was initially compared with each model in isolation. The findings demonstrated that our approach could successfully classify intrusion with high values for the F1-score, accuracy, recall and precision metrics. Next, our model was evaluated on the NSL-KDD dataset using confusion matrices, and it was shown to generate better results than the alternative models for all types of intrusion apart from U2R. Finally, a comparison between our architecture and state-of-the-art alternatives was performed, and results revealed that the proposed method was superior to the other methods.

**Reviewer#1, Concern # 3:** Authors should explain the feature selection algorithms used in the work.

**Author Response:** We would like to thank the reviewer for comments. In fact, the feature extraction/selection process of our proposed method is intended to be explained in the Proposed Method Section and, CNN Arhitectures subsection of the Prior Schemes Used In the Suggested Method Section. Our proposed novel framework for intrusion detection is introduced in proposed method section in our manuscript. Our framework leverages deep learning algorithms and incorporates a hybrid DNN architecture that supports hyperparameter optimisation. The most important contribution of this framework is its unique hash architecture, which effectively combines three commonly used pre-trained network models and enables hyperparameter optimisation. The methodology of our system consists of five main phases. First, intrusion detection data are collected from two extensive datasets. The data are then subjected to pre-processing, as described in depth in the subsection on data pre-processing. Thirdly, high- and low-level intrusion detection features are extracted using pre-trained networks in the feature extractor phase, which also involves performing hyperparameter optimisation. At this stage, the ResNet50, GoogLeNet and AlexNet deep learning architectures are used for feature extraction/selection process, while the PSO method is used for hyperparameter optimization. Aforementioned deep learning architectures are explanied detailed in the CNN Arhitectures subsection of the Prior Schemes Used In the Suggested Method Section. Next, the proposed architecture is trained using the optimised hyperparameters obtained through PSO together with the intrusion detection datasets in the fully connected layers. Finally, the output phase involves classification using a SoftMax classifier.

**Author Action:** In addition to the previously added sentences in manuscript, the Proposed Method Section of manuscript has been updated in manuscript as shown below so as to make the proper clarification in this section. Details were shown at a marked-up copy of our manuscript (revised manuscript with track changes).

For the Proposed Method Section:

A novel framework for intrusion detection is introduced in this section. Our framework leverages deep learning algorithms and incorporates a hybrid DNN architecture that supports hyperparameter optimisation. The most important contribution of this framework is its unique hash architecture, which effectively combines three commonly used pre-trained network models and enables hyperparameter optimisation. The methodology of our system consists of five main phases, as shown in Figure 4. First, intrusion detection data are collected from two extensive datasets. The data are then subjected to pre-processing, as described in depth in the subsection on data pre-processing. Thirdly, high- and low-level intrusion detection features are extracted using pre-trained networks in the feature extractor phase, which also involves performing hyperparameter optimisation. At this stage, the ResNet50, GoogLeNet and AlexNet deep learning architectures are used for feature extraction/selection process, while the PSO method is used for hyperparameter optimization. Next, the proposed architecture is trained using the optimised hyperparameters obtained through PSO together with the intrusion detection datasets in the fully connected (FC) layers. Finally, the output phase involves classification using a SoftMax classifier.

For the CNN Arhitectures subsection of the Prior Schemes Used In the Suggested Method Section:

ResNet-50 is a CNN with a depth of 50 layers. The goal of the ResNet model is to resolve the problem of a decrease in performance with CNNs, which is done by adding shortcuts called residual learning blocks between the layers. These blocks, illustrated in Figure 8(a), can be considered the core structural components of ResNet. In a residual block, the path that results from adding the input x directly to the network’s output is referred to as a shortcut or jump link. The ResNet50 architecture, as shown in Figure 8(b), has 25.6 million parameters, and includes a SoftMax layer, two pooling operations, an FC layer and five convolutional blocks.

GoogLeNet is a CNN with seven million process parameters and a depth of 27 layers, including pooling layers. It is also known as Inception, and is a deep CNN architecture developed by researchers at Google. It includes Inception modules, which use multiple convolutional filter sizes in parallel to capture features at different scales. This module consists of a shortcut branch and few deeper branches, and it provides the width item in the model to be obtained. The architecture of GoogLeNet [57], illustrated in Figure 9, aims to strike a balance between depth and computational efficiency by reducing the number of parameters. The architecture consists of four main sections: the output classifier, stacked, stem, and Inception auxiliary classifiers modules. In addition, this architectur e has 144 layers, including FC, convolution, max-pooling, output, SoftMax, input layers and ReLUs, and it contains nine Inception modules.

AlexNet is a groundbreaking deep CNN architecture that played a crucial role in popularising deep learning for computer vision tasks. Developed by Alex Krizhevsky et al., it achieved remarkable success by winning the ImageNet Large Scale Visual Recognition Challenge in 201268. This network, as shown in Figure 10, consists of seven ReLUs, two normalisation layers and three pooling layers following the convolution layers. In addition, it includes a SoftMax layer and FC layers for classification and learning.

**Reviewer#1, Concern # 4:** The paper does not explicitly address the issue of class imbalance in the training data. Imbalanced datasets can lead to biased models, and it's important to understand how the proposed research handles this issue.

**Author Response:** Thank you for the comment we do agree and have made changes accordingly. The following statements, shown in Author Action section, is added in the abstract of manuscript.

**Author Action:** After hyperparameter tuning has been carried out, training is conducted in order to achieve a high accuracy rate. In this stage, public comprehensive datasets mentioned above and the PSO method, which is a metaheuristic method and whose details are explained in the Proposed Method and Prior Schemes Used In the Suggested Method Sections, were used to minimize the problem of class imbalance in the training data and obtain high accuracy values. In addition, 20% of the intrusion data are used for testing, 10% for validation, and 70% for training.

**Reviewer#1, Concern # 5:** The related work section is very small, an updated and complete literature review should be conducted. Some latest papers listed below which studied similar problems can be discussed: 1). Feature selection in intrusion detection systems: a new hybrid fusion of Bat algorithm and Residue Number System; 2). XIDINTFL‑VAE: XGBoost‑based intrusion detection of imbalance network traffic via class‑wise focal loss variational autoencoder. 3). Modified genetic algorithm and fine-tuned long short-term memory network for intrusion detection in the internet of things networks with edge capabilities.

**Author Response:** Thank you for the comment we do agree and have made changes accordingly. The following statements, shown in Author Action section, is added in the Related Work and References sections of our manuscript.

**Author Action:**

For the Related Work Section:

Saheed et al. [56] introduces a hybrid feature selection approach combining the Bat metaheuristic algorithm with the Residue Number System (RNS) to enhance intrusion detection systems (IDS). The Bat algorithm is used to identify significant features by optimizing search spaces, while RNS improves computational speed and reduces complexity through its modular arithmetic properties. Principal Component Analysis (PCA) is applied for feature extraction, and classification is performed using Naïve Bayes (NB) and K-Nearest Neighbors (KNN) algorithms. Experimental evaluations on the NSL-KDD dataset demonstrated the superior performance of the Bat-RNS+PCA+KNN model, achieving a detection high accuracy, precision and F-Score values.

Abdulganiyu et al. [57] developed XIDINTFL-VAE model to detect minority class intrusions to detect minority class intrusions in highly imbalanced network traffic. This framework combines Class-Wise Focal Loss (CWFL) with a Variational AutoEncoder (VAE) and integrates XGBoost as the classifier. The CWFL-VAE generates synthetic data tailored to the minority classes, improving the detection capabilities for rare and critical attacks. XGBoost is used for robust classification, benefiting from its ability to handle structured data and imbalances. Experiments were conducted on the NSL-KDD and CSE-CIC-IDS2018 datasets, demonstrating significant improvements over traditional methods such as SMOTE and ADASYN, as well as existing classifiers like Logistic Regression and Random Forest. The XIDINTFL-VAE achieved high performance across metrics, with obtained high precision, F1 score and recall values, effectively balancing detection accuracy and minimizing false alarms.

Saheed et al. [58] proposes the IoT-Defender framework, a lightweight intrusion detection system (IDS) designed to enhance IoT network security using edge computing. IoT-Defender integrates a Modified Genetic Algorithm (MGA) for optimal feature selection and a Fine-Tuned Long Short-Term Memory (LSTM) model for anomaly detection. The framework employs MGA to identify the most relevant features from IoT-specific datasets (BoT-IoT, UNSW-NB15, and N-BaIoT) and uses a genetic algorithm to fine-tune the LSTM architecture, optimizing parameters like the number of hidden layers and learning rates. The system also incorporates a focal loss function to address class imbalance in IoT traffic data. Experimental results on edge devices such as the Raspberry Pi 4 show that IoT-Defender has a 2.56% It shows that it achieves a low false alarm rate and achieves high accuracy and detection rate.

For the References Section:

[56] Saheed, Y. K., Kehinde, T. O., Ayobami Raji, M., & Baba, U. A. (2023). Feature selection in intrusion detection systems: a new hybrid fusion of Bat algorithm and Residue Number System. Journal of Information and Telecommunication, 8(2), 189–207. https://doi.org/10.1080/24751839.2023.2272484

[57] Abdulganiyu, O.H., Tchakoucht, T.A., Saheed, Y.K. et al. XIDINTFL-VAE: XGBoost-based intrusion detection of imbalance network traffic via class-wise focal loss variational autoencoder. J Supercomput 81, 16 (2025). https://doi.org/10.1007/s11227-024-06552-5

[58] Saheed, Y.K., Abdulganiyu, O.H., Tchakoucht, T.A., 2024. Modified genetic algorithm and fine-tuned long short-term memory network for intrusion detection in the internet of things networks with edge capabilities. Appl. Soft Comput. 111434.

**Reviewer#1, Concern # 6:** In the end of related work section, highlight in 10-15 lines what overall technical gaps are observed in existing techniques that led to the design of the proposed methodology.

Author Response**: Thank you for the comment we do agree and have made changes accordingly. The following** statements, shown in Author Action section, is added in the related work section of manuscript.

**Author Action:** Even though a lot of research has been done on intrusion detection, it is still quite challenging to detect different kinds of network intrusions effectively in the field of cybersecurity. Cyberattacks are also growing and changing at exponential rates, and many of these modern cyberattacks cannot yet be detected by any reliable system or technique. Additionally, traditional and contemporary IDSs are no longer able to differentiate sophisticated intrusions from typical network traffic. Therefore, a novel hybrid deep learning architecture for the effective detection of many intrusion types has been developed in this study. The proposed architecture is a hybrid of three pre-trained networks based on transfer learning. Our work's primary contribution is a novel deep learning-based hybrid architecture that combines three well-known pre-trained network models in an optimized manner while using PSO for hyperparameter optimization.

**Reviewer#1, Concern # 7:** Although the English is generally quite good, there are quite a few minor grammatical errors, and a careful read-through is needed to eliminate these errors. The spelling mistake should be corrected by reading through the manuscript.

**Author Response:** Thank you for the comment, we do agree with the comment.

**Author Action:** After final improvements, it was proofread by a professional editor in order to remove typos and grammatical errors, as you can observe from the text. The certificate regarding this is available below.
